# Corticospinal Intermittent Theta Burst Stimulation Propelling Sensorimotor Function Recovery in Complete Spinal Cord Injury: Protocol for a Randomized Controlled Trial

**DOI:** 10.2196/66531

**Published:** 2025-06-27

**Authors:** Deeksha Patel, Rohit Banerjee, Kamran Farooque, Deepak Gupta, Bhavuk Garg, Nand Kumar, Kanwal Preet Kochhar, Suman Jain

**Affiliations:** 1 Department of Physiology All India Institute of Medical Sciences, New Delhi Delhi India; 2 Department of Orthopedics All India Institute of Medical Sciences, New Delhi Delhi India; 3 Department of Neurosurgery All India Institute of Medical Sciences, New Delhi New Delhi India; 4 Department of Psychiatry All India Institute of Medical Sciences, New Delhi Delhi India

**Keywords:** American Spinal Injury Association Impairment Scale, excitatory-inhibitory circuitry, intermittent theta burst stimulation, physical rehabilitation, protocol, randomized control trial, spinal cord injury, synaptic plasticity

## Abstract

**Background:**

Intermittent theta burst stimulation (iTBS) is a noninvasive stimulation technique to induce neuronal and synaptic plasticity. The induced cortical plasticity is imperative in the recovery of motor and sensory functions. Spinal cord injury (SCI) causes damage to neurons and results in sensorimotor dysfunction. The effect of iTBS on recovery of motor and sensory dysfunction in complete SCI (cSCI) is still elusive.

**Objective:**

This study aims to assess the effect of iTBS on corticospinal tract integrity, plasticity, and regaining of motor and sensory function in patients with cSCI. The rationale behind using an iTBS protocol is to modify and augment the communication between spared neurons of the corticospinal tract and strengthen the synaptic transmission, which will improve motor function in underlying muscles.

**Methods:**

A total of 40 patients with cSCI with American Spinal Injury Association (ASIA) grade A, aged 18-60 years, were randomly assigned to 5 groups. To evaluate the efficacy of iTBS versus traditional repetitive transcranial magnetic stimulation, patients were categorized into placebo, repetitive transcranial magnetic stimulation, and iTBS. In addition, to determine the optimal site for stimulation, groups were further subdivided into motor cortex, spinal cord, and combined. Each patient underwent 10 iTBS sessions twice daily for 5 consecutive days. Neurological outcomes and functional outcome parameters will be assessed. Electrophysiological evaluations included transcranial magnetic stimulation single-pulse and paired-pulse parameters. The effect of iTBS intervention on biomarkers will be quantified using the enzyme-linked immunosorbent assay, while neurotransmitters will be quantified by liquid chromatography and tandem mass spectrometry. Measurements will be done before and after the intervention, with follow-ups at 1, 2, and 3 months.

**Results:**

The outcome of the study will be defined by electrophysiological parameters elicited by single- and paired-pulse stimulation, ASIA score, pain, activities of daily life, quality of life, anxiety, depression, and biomarkers related to SCI. The results of this study will uncover the effectiveness of iTBS stimulation on (1) recovery of motor and sensory function in cSCI, (2) excitability of the corticospinal tract, (3) neurological recovery and modulation of pain, and (4) cortical reorganization after injury.

**Conclusions:**

iTBS in conjunction with an individualized rehabilitation program may serve as an integrated strategy to rejuvenate locomotor abilities and improve the overall quality of life for people with cSCI.

**Trial Registration:**

Clinical Trials Registry- India CTRI/2022/11/047038; https://ctri.nic.in/Clinicaltrials/main1.php?EncHid=13361.98443

**International Registered Report Identifier (IRRID):**

DERR1-10.2196/66531

## Introduction

### Background

Spinal cord injury (SCI) is a neurological condition that causes progressive neurodegeneration. The major symptoms of SCI are paralysis, paresthesia, pain, spasticity, bladder, bowel, and sexual dysfunction. It largely affects the young population (21-49 years); thus, the psychological impact on a healthy individual to adapt to a paraplegic or quadriplegic condition in their early life is devastating. SCI causes damage to neurons, nerves, and other surrounding cells that send and receive signals from different body parts to the brain via the spinal cord (SC) and vice versa [1]. SCI can be traumatic or nontraumatic. Traumatic SCI is a great challenge for therapeutic management considering its high morbidity. There is complete or incomplete loss of locomotor, sensory, and autonomic functions depending on the severity and level of the lesion [2]. The consequences of injury are not just a break in communication between neurons, but a cascade of events that sets up a vicious cycle and leads to widespread neuronal degeneration, cell death, and the formation of glial scars [3]. Cellular components such as proteins, phospholipids, neurotransmitters, and metabolites derived from SC neurons and glial cells diffuse from the injury site into the cerebrospinal fluid and blood [4]. These biomarkers could serve as good diagnostic markers to predict the severity of the injury. To regain functional connectivity, and attenuate gliosis and secondary injury, activity-dependent strategies have been proposed to be quite effective. The current treatment modalities for SCI include surgery, pain management, and rehabilitation. However, functional recovery by restoring connections of the corticospinal tract after a complete SCI is challenging.

High-frequency repetitive transcranial magnetic stimulation (rTMS) has decreased neuropathic pain and spasticity in incomplete SC–injured patients [5,6]. A newer form of patterned transcranial magnetic stimulation (TMS), intermittent theta burst stimulation (iTBS) is now being studied for the improvement of symptoms in incomplete SCI [7]. There is very limited literature available, showing the effect of either cortical iTBS or trans-spinal TMS in combination with a rehabilitation program to promote repair, regeneration, and recovery in patients with complete SCI (cSCI). This study aims to determine the functional outcomes of administering iTBS at the cortex as well as at SC along with intensive rehabilitation programs in patients with cSCI.

### Study Objectives

This study aims to evaluate the comparative efficacy of iTBS and rTMS in promoting motor function recovery in individuals with cSCI. In addition, it seeks to identify the most effective stimulation site—motor cortex, SC, or a combined approach—for optimizing therapeutic outcomes. The study will further assess the impact of these TMS protocols along with customized rehabilitation programs on sensory and locomotor function, cortical excitability and neuroplasticity, SCI-related biomarkers, and self-reported outcomes, including pain, anxiety, depression, and overall quality of life.

## Methods

### Study Design

A double-blinded, prospective, randomized, placebo-controlled study will be conducted. The computer-generated sequence will be sealed in sequentially numbered envelopes, which will be opened after the patient is enrolled in the study. Both the patient and investigator will be blinded to the intervention. An experienced therapist will perform the intervention protocol. This protocol is based on the SPIRIT (Standard Protocol Items: Recommendations for Interventional Trials) guidelines (checklist provided in Multimedia Appendix 1).

### Duration of the Study

The recruitment, intervention, and follow-up phase will be 2 years, followed by 6 months of data analysis. Patient recruitment will start in January 2023 and the trial will be completed by July 2025.

### Inclusion and Exclusion Criteria

Adults aged 18 to 60 years with a thoracolumbar (T1-L5) SCI and complete motor loss below the lesion level (American Spinal Injury Association [ASIA] score A) will be screened and recruited within 1 month of injury.

Exclusion criteria include patients with osteoporotic fractures, a history of neurological or orthopedic diseases affecting the SC, head injuries, ferromagnetic metallic implants near the target stimulation area, pacemakers, cognitive impairment, pregnancy, a history of seizures, or acute eczema or bedsores.

### Confidentiality

The personal privacy of patients will be protected. The results of this study will not disclose any identifying and personal information of the patient without his or her permission.

### Sample Size

The sample size for this study is determined based on the methodology outlined by Roy et al [8], which assessed the reduction in motor evoked potential (MEP) amplitude following intervention. Using an α level of 0.05 and a power of 90%, these parameters are incorporated into the 2-tailed formula for sample size calculation.

For a 2-sample comparison of means, the null hypothesis (H0: m1=m2) will tested, where m1 and m2 represent the means of 2 distinct populations. The assumptions for the calculation are as follows:

α=.05 (2-sided), Power=0.90, m1=12.85, m2=13.53, SD1=0.3, SD2=0.3, n2/n1=1.00

Based on these assumptions, the estimated required sample size is calculated to be 6 subjects per group. To account for potential dropouts, a 20% adjustment is applied, resulting in a revised sample size of 8 participants per group. Consequently, the total minimum sample size required for the study is 40 participants.

### Experimental Groups

A total of 40 patients will be randomized into 5 groups using computer-generated random numbers. Patients will be categorized into placebo, rTMS, and iTBS to evaluate the efficacy of iTBS versus traditional rTMS on the primary motor cortex (MC). To determine the optimal site for iTBS application, stimulation was given at either MC or SC or combined MC+SC.

### Mode of Stimulation

Group classification based on the mode of stimulation is listed as follows:

- Group 1: Placebo
- Group 2: High-frequency rTMS
- Group 3: iTBS

### Site of Stimulation

Group classification based on the site of stimulation is listed as follows:

- Group 3: iTBS on motor cortex
- Group 4: iTBS on the SC
- Group 5: iTBS on the motor cortex and SC

**Figure 1:**
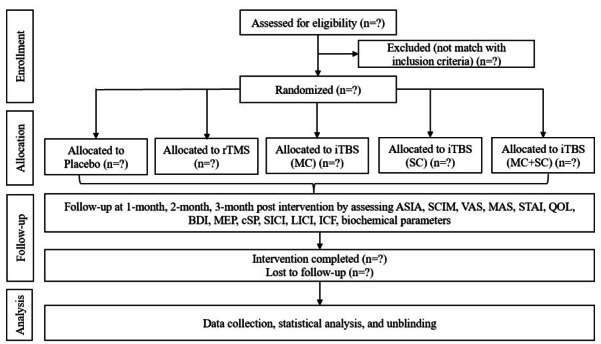
CONSORT (Consolidated Standards of Reporting Trials) flow diagram. ASIA: American Spinal Injury Association; BDI: beck depression inventory; cSP: cortical silent period; ICF: intracortical facilitation; iTBS: intermittent theta burst stimulation; LICI: long-interval intracortical inhibition; MAS: ___; MC: motor cortex; MEP: motor evoked potential; QOL: quality of life; rTMS: repetitive transcranial magnetic stimulation; SC: spinal cord; SCIM: Spinal Cord Independence Measure; SICI: short-interval intracortical inhibition; STAI: State-Trait Anxiety Inventory; VAS: Visual Analog Scale.

This trial’s conduct and report will follow the CONSORT (Consolidated Standards of Reporting Trials) statement for randomized trials (Figure 1).

### Patient Recruitment and Parameter Recording

**Figure 2:**
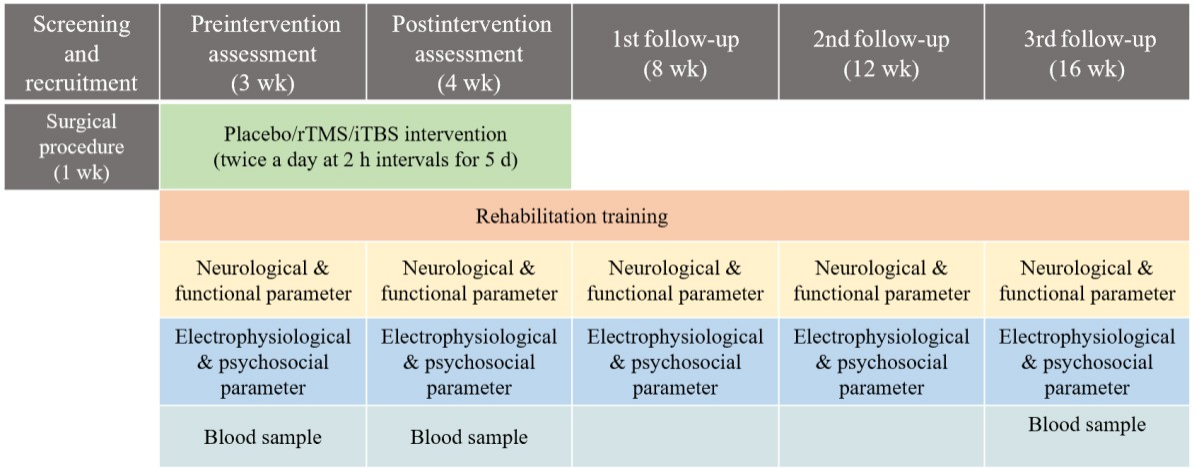
Timeline: all the intervention and recording paradigms are organized as a timeline. iTBS: intermittent theta burst stimulation; rTMS: repetitive transcranial magnetic stimulation.

Participants will be enrolled from the Jai Prakash Narayan Apex Trauma Centre at AIIMS (All India Institute Of Medical Sciences) Delhi. Following SCI surgery, a 15-day period will be designated for vertebral stabilization, after which sutures will be removed on the 15th day. Once the sutures are removed, baseline parameters will be measured. Patients will then be randomly assigned to one of the intervention groups. After completing the intervention, postintervention parameters will be recorded and follow-up assessments will be conducted at 1, 2, and 3 months (Figure 2). To ensure consistency during the intervention, all assessments will be recorded by the blinded primary investigator.

### Intervention Protocol

During the intervention phase, patients will be randomly allocated to one of the interventional groups. The rTMS or iTBS will be administered using the Neurosoft - Neuro-MS 5 device (Neurosoft Ltd), a commercially available transcranial magnetic stimulator. The device will be equipped with both an angulated figure 8–shaped coil and a circular coil for stimulation. The intervention (rTMS or iTBS) will be applied to the lower-limb motor area located in M1 (to stimulate both lower limbs), with the handle of the coil aligned parallel to the interhemispheric midline (pointing occipitally) based on the vertex position according to the International 10-20 EEG system. For SC stimulation, the circular coil will be positioned over the injury site. In the placebo stimulation group, a sham coil (not generating a magnetic field but producing similar click sounds) will be used and the stimulation protocol remains akin to the iTBS group.

The standard protocol for iTBS will be used: 3-pulse bursts at 50 Hz, repeated at 5 Hz, with a 2-second train repeated every 10 seconds for 20 repetitions, amounting to a total of 600 pulses [7]. The rTMS protocol will consist of 1600 pulses at a frequency of 20 Hz, with a 2-second train repeated every 30 seconds for 20 minutes [9]. A resting motor threshold (RMT) of 90% will be used for both rTMS and iTBS stimulation intensity. RMT will be determined by recording MEP from the abductor pollicis brevis (APB). RMT will be recorded by placing figure-eight coils over the motor hotspots of the APB. The minimum stimulus intensity that causes an MEP with a peak-to-peak amplitude of at least 50 µV in at least 50% of consecutive stimuli will be considered as RMT. Interventions will be administered for 5 consecutive days, twice daily, with a total of 10 sessions [10].

### Postintervention Care

All the participants will be required to fill out a TMS safety questionnaire both before and after the intervention. Briefly, the preintervention questionnaire takes care of any implants, past history of seizures, pregnancy, or head trauma, whereas the postintervention questionnaire reports any adverse effects observed by patients like headache, nausea, ear discomfort, or any seizure. Besides these, the participants will also undergo thorough screening for contraindications, continuous monitoring during and after stimulation, and adherence to standardized emergency protocols as approved by the institutional review board. If a patient experiences an adverse event during the trial, the principal investigator will provide treatment and corresponding financial compensation. Patients who are enrolled in the placebo group will also receive the conventional rehabilitation program during the study.

### Outcomes

#### Primary Outcome (Neurological)

The primary outcome will measure the severity of injury with the ASIA Scoring System. The ASIA impairment score (AIS) ranges from complete loss of sensation and movement (AIS=A) to normal neurological function (AIS=E). The ASIA motor score uses a test of the strength of 10 key muscles on each side of the body (eg, elbow flexors, wrist extensors, hip flexors, quadriceps, and dorsi flexors). The score ranges from 0 (no contraction) to 5 (normal resistance) through a full range of motion. A total possible score of 50 for the upper extremities and 50 for the lower extremities may be obtained [11]. The ASIA sensory score involves pinprick and light touch sensation at key points representing each dermatome of the body, scored on a 3-point scale (0, 1, and 2). Scores are summed to give a total possible score of 224, where a higher score indicates better sensation than a lower score.

#### Secondary Outcomes

##### Functional Parameter Outcomes

Walking Index for Spinal Cord Injury II (WISCI-II) is a 0-20 level scale that evaluates the walking activity of a patient based on physical assistance, the need for braces and walker, and other adaptive devices. The levels on the scale are scored from 0 (patient unable to walk) to 20 (patient walking without braces and adaptive devices and without any physical assistance for at least 10 m) [12].

Spinal Cord Independence Measure-III (SCIM-III) includes 19 tasks organized in 3 subclasses based on the patient’s general activity: self-care (score 0-20), respiration and sphincter management (score 0-40), and mobility (score 0-40). The overall scores range from 0 to 100, where a 0 score defines a total dependence of the patient on the caregiver and a score of 100 indicates complete independence [13].

##### Electrophysiological Parameter Outcomes

###### Single-Pulse TMS

A single pulse of TMS at minimum stimulus intensity will be delivered at the motor cortex that elicits an MEP of ≥ 50 µV at least 5 out of 10 trials in the target muscle (APB) and will be recorded as RMT [14]. For recording the inhibitory activity, a single TMS is delivered to produce an interruption in ongoing EMG activity during a tonic contraction followed by the reoccurring EMG activity. The duration of silencing of EMG in response to TMS will be measured as a cortical silent period (cSP) [15]. The recruitment curve plotted between the TMS intensity (%) and MEP sizes.

###### Paired-Pulse TMS

A total of 2 TMS pulses, conditioning stimulus, and test stimulus, delivered at specific interstimulus intervals result in either facilitation or inhibition. In short-interval intracortical inhibition, MEP elicited by test stimulus is inhibited when preceded by a conditioning stimulus at ~1- to 5-millisecond intervals. In long-interval intracortical inhibition, the interstimulus interval is kept between 50 and 200 milliseconds, whereas in intracortical facilitation, the interval is ~10-30 milliseconds [15].

##### Psychosocial Parameter Outcomes

Visual Analog Scale (VAS) measures the perceived intensity of pain on a self-explanatory scale of 0 to 10, where 0 (no pain) to 10 (severe pain) [16].

The Beck Depression Inventory-II (BDI-II) is a 21-question multiple-choice self-report inventory to measure the presence and severity of depression. The cut-off scores with 0-9 indicating normal, 10-19 indicating mild depression, 20-30 indicating moderate depression, and 31-63 indicating severe depression [17].

The State-Trait Anxiety Inventory (STAI) is a 40-item self-report scale, commonly used to measure anxiety [18].

The World Health Organization Quality of Life-BREF (WHOQOL-BREF) Scale was developed in the context of the 4 domains defining the QOL: physical, psychological, social, and environmental. The higher the QOL score, the higher the life satisfaction [19].

##### Biochemical Quantification

The disruption of the blood-spinal cord barrier and secondary damage following the injury releases several chemokines, growth factors, and neurotransmitters in cerebrospinal fluid and systemic circulation, which could help predict the severity of the injury. The released components, such as myelin basic protein (MBP), interleukins, phosphorylated neurofilaments, brain-derived neurotrophic factor, Fas-ligand, gamma-aminobutyric acid, and glutamate, may serve as potential biomarkers for remyelination, inflammation, neuronal survival, and excitatory or inhibitory neurotransmitters after SCI [20-23]. Liquid chromatography and tandem mass spectrometry and enzyme-linked immunosorbent assays will be performed to quantify these biomarkers in plasma and serum samples.

##### Physiotherapy

**Figure 3:**
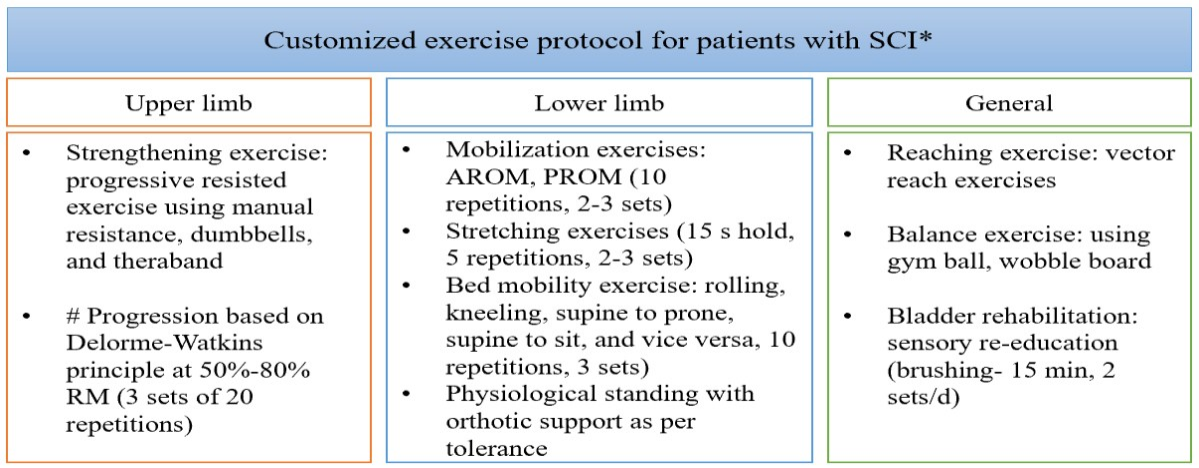
Outline of physical rehabilitation regime for patients with spinal cord injury. Exercise prescription and progression will be based on the individual’s performance and tolerance to baseline exercises. AROM: active range of motion; PROM: passive range of motion; RM: repetition maximum; SCI: spinal cord injury.

A trained physiotherapist will be assigned to provide individualized rehabilitation training for 5 days. After this period, patients will be instructed to perform the exercises independently at home, maintaining a logbook and submitting weekly videos of their performance for evaluation by the physiotherapist till the time of last follow-up (Figure 3).

#### Statistical Analysis

Data will be analyzed using STATA 14.0 software (StataCorp). Continuous variables will be presented as mean and SD, and categorical variables as frequencies and percentages. The primary analysis will be the intention-to-treat analysis, where baseline values will be carried forward for subjects with missing data. Unless stated otherwise, intention-to-treat data will be presented throughout the study. In addition, a per-protocol analysis will be conducted, including only participants who comply with the protocol.

Normality will be evaluated using the Shapiro-Wilk test for each outcome, homogeneity of variance will be checked using the Levene test, and the Mauchly test will be used for sphericity. Depending on the data distribution, parametric or nonparametric tests will be applied.

If the data are found to be uniformly distributed, we will use repeated measures ANOVA to assess changes over time within and between groups. Bonferroni or Tukey correction will be done for post hoc pairwise comparisons. Nonparametric tests like the Friedman test followed by appropriate post hoc tests will be used for skewed data. Corrections for multiple comparisons will be applied as specified to reduce the risk of type I errors. To analyze categorical variables, the chi-square or Fisher exact test will be used to assess associations between groups.

A *P* value less than .05 with a 95% CI will be considered statistically significant.

#### Ethical Considerations

Ethical approval has been obtained from the Institutional Ethical Committee of All India Institute of Medical Sciences, Delhi (Project Ref. Id: IECPG/551/7/2022). The study is registered in the Clinical Trials Registry- India (CTRI; reference number CTRI/2022/11/047038). Informed, voluntary, and written consent will be taken, and the participants will be given a choice to withdraw from the study at any given point in time. They will be duly informed of the duration of the study along with any potential risks or side effects associated with TMS intervention. If a patient experiences an adverse event during the trial, the investigators will provide treatment and corresponding financial compensation. No names or any other forms of identification will be used in publications or reports resulting from the study without the permission of the participants.

All the experiments will be carried out in the Brain Stimulation and Neuromodulation Laboratory, Department of Physiology, and CARE, Department of Psychiatry, AIIMS, New Delhi.

## Results

This randomized controlled trial will assess the impact of iTBS on motor and sensory functional recovery, alterations in corticospinal excitability, biomarkers, and quality of life in patients with cSCI. The project was funded by DBT in July 2019. The randomized controlled trial has been registered with the Clinical Trials Registry-India (CTRI/2022/11/047038) and ethical approval obtained from the Institutional Ethics Committee, AIIMS, Delhi (Project Ref. Id: IECPG/551/7/2022). The project was initiated in January 2023 and is likely to be completed by June 2025. The results of this study will be analyzed from January to July 2025 and will be submitted for publication in peer-reviewed scientific journals. We presume this study may support integrating iTBS into a rehabilitation program for motor and sensory recovery in patients with cSCI.

## Discussion

### Principal Findings

SCI significantly impairs the corticospinal integrity and afferent-efferent input-output circuitry. Theoretically, the pyramidal tract is the primary neural pathway that links the cortex and SC to facilitate the movement of distal extremities. The primary purpose of any treatment is to reconstruct the neural circuit immediately after SCI for functional sensory-motor recovery. To facilitate neural circuit reconstruction, it is required to stimulate nerve cell sprouting and regeneration as well as increase the strength of the existing neuronal connections. Previous research demonstrated that individuals with incomplete SCI can benefit from locomotor training to enhance their motor skills. Rehabilitation programs may use learning and relearning mechanisms, uncovering a previously inactive synapse, and forming a new synapse [24]. However, the reconstruction of the damaged neural circuit is quite difficult with locomotor training alone. Studies have shown that the functional effects of exercise along with transcranial magnetic stimulation, can activate spared neural pathways and enhance the possibility of neural reconstruction [25,26].

TMS induces electrical currents in underlying cortical areas, depolarizes neurons, and generates an action potential that modulates the activity of spinal motor neurons and target muscles via the corticospinal tract. Repetitive transcranial magnetic stimulation as well as iTBS is an intervention in various psychiatric and pain conditions. In SCI, few studies suggest improvement in locomotor function, spasticity, and pain in incomplete patients [6]. Although, the functional mechanism of rTMS or iTBS on sensorimotor recovery in patients with SCI is not fully understood, but thought to induce synaptic plasticity via LTP or LTD-like effects [27], thereby promoting functional recovery in patients with SCI.

In addition, SC stimulation can modulate the activity of the local central pattern motor generators, which promotes synaptic strengthening [28]. In rat models of complete and incomplete SCI, a significant attenuation of glial scaring, lesion volume, neurotransmitter imbalance, muscle atrophy, and facilitation of neuronal survival, axonal regeneration, and myogenesis has been shown following whole-body magnetic field exposure [29,30]. Therefore, we propose that SC stimulation along with motor cortical stimulation would attenuate secondary damage and promote regeneration even in patients with cSCI, leading to long-term functional recovery.

Various single- and paired-pulse paradigms of TMS have been used for the assessment of cortical excitability, plasticity, integrity of the corticospinal tract, and excitatory-inhibitory neural circuitry of the motor cortex. We shall use all paired-pulse paradigms (short-interval intracortical inhibition, intracortical facilitation, and long-interval intracortical inhibition) to objectively assess excitatory-inhibitory neural circuitry and single-pulse paradigms (RMT, MEP, recruitment curves, and contralateral silent period) for unraveling the excitability of corticospinal circuitry and motor units.

### Limitations

While this study offers valuable insights, several limitations must be acknowledged that may affect the interpretation and broader application of the findings. First, the sample size is relatively small, which could reduce the statistical power and may not fully represent the broader population of individuals with SCIs. Second, variability in the severity of SC injuries among participants may influence the outcomes, as responses to treatment could differ based on the level and completeness of the injury. Finally, the short duration of the follow-up period may not adequately capture the long-term effects of the interventions, suggesting the need for longer-term studies to assess the sustainability of the benefits observed.

### Conclusion

An intensive individualized rehabilitation regime coupled with iTBS could be a holistic management strategy that can restore locomotor function and quality of life in patients with cSCI. TMS is also a promising tool to evaluate the cortical plasticity and excitability in SCI and thereby understand the mechanism of action of the proposed intervention and characterize the effective connectivity of neural circuits and mechanisms regulating the balance between inhibition and facilitation within the corticospinal pathway.

## Appendix

Multimedia Appendix 1 SPIRIT (Standard Protocol Items: Recommendation for Interventional Trials) checklist.

## Abbreviations

AIIMS: All India Institute Of Medical Sciences
AIS: ASIA impairment score
APB: abductor pollicis brevis
ASIA: American Spinal Injury Association
BDI-II: Beck Depression Inventory-II
CONSORT: Consolidated Standards of Reporting Trials
cSCI: complete spinal cord injury
CTRI: Clinical Trials Registry- India
iTBS: intermittent theta burst stimulation
MBP: myelin basic protein
MC: motor cortex
MEP: motor evoked potential
RMT: resting motor threshold
rTMS: repetitive transcranial magnetic stimulation
SC: spinal cord
SCI: spinal cord injury
SCIM-III: Spinal Cord Independence Measure-III
SPIRIT: Standard Protocol Items: Recommendations for Interventional Trials
STAI: state-Trait Anxiety Inventory
TMS: transcranial magnetic stimulation
VAS: Visual Analog Scale
WHOQOL-BREF: World Health Organization Quality of Life-BREF
WISCI-II: Walking Index for Spinal Cord Injury II

## Data Availability

Data sharing is not applicable to this article as no data sets were generated or analyzed during this study.
