# Supplementary material for: Corticospinal Intermittent Theta Burst Stimulation Propelling Sensorimotor Function Recovery in Complete Spinal Cord Injury: Protocol for a Randomized Controlled Trial
Source: JMIR Res Protoc. 2025 Jun 27;14:e66531. doi: 10.2196/66531 (PMC12254704; doi:10.2196/66531)
Supplement: Multimedia Appendix 1 [file resprot_v14i1e66531_app1.docx]

| ASSESSMENT | | Before surgery | Preintervention | Intervention | Postintervention | 1st Follow-up | 2nd Follow-up | 3rd Follow-up |
| --- | --- | --- | --- | --- | --- | --- | --- | --- |
| Enrolment | | √ |  |  |  |  |  |  |
| Consent | | √ |  |  |  |  |  |  |
| Screening | | √ |  |  |  |  |  |  |
| Demographic details | | √ |  |  |  |  |  |  |
| Vitals | | √ | √ |  | √ | √ | √ | √ |
| Medical history | | √ |  |  |  |  |  |  |
| Inclusion | | √ |  |  |  |  |  |  |
| Exclusion | | √ |  |  |  |  |  |  |
| Randomization | |  | √ |  |  |  |  |  |
| Blinding | |  | √ |  |  |  |  |  |
| Blood sample collection | |  | √ |  | √ | √ | √ |  |
| ASIA-motor | |  | √ |  | √ | √ | √ | √ |
| ASIA-sensory | |  | √ |  | √ | √ | √ | √ |
| WISCI | |  | √ |  | √ | √ | √ | √ |
| MAS | |  | √ |  | √ | √ | √ | √ |
| SCIM | |  | √ |  | √ | √ | √ | √ |
| VAS | |  | √ |  | √ | √ | √ | √ |
| WHOQOL | |  | √ |  | √ | √ | √ | √ |
| BDI-II | |  | √ |  | √ | √ | √ | √ |
| STAI | |  | √ |  | √ | √ | √ | √ |
| TMS pre-therapy questionnaire | |  | √ |  |  |  |  |  |
| Interventions | |  |  |  |  |  |  |  |
|  | Placebo |  |  | √ |  |  |  |  |
|  | Real- rTMS |  |  | √ |  |  |  |  |
|  | Real - ITBS |  |  | √ |  |  |  |  |
| TMS Parameters | |  | √ |  | √ | √ | √ | √ |
|  | RMT |  | √ |  | √ | √ | √ | √ |
|  | MEP |  | √ |  | √ | √ | √ | √ |
|  | cSP |  | √ |  | √ | √ | √ | √ |
|  | SICI |  | √ |  | √ | √ | √ | √ |
|  | LICI |  | √ |  | √ | √ | √ | √ |
|  | ICF |  | √ |  | √ | √ | √ | √ |
| TMS post-therapy questionnaire | |  |  |  | √ |  |  |  |
| Rehabilitation programme | |  |  | √ | √ | √ | √ | √ |
| Unblinding | |  |  |  |  |  |  | √ |
